# Prevalence, incidence and risk factors for rugby-related injuries: A survey of the Safari Sevens tournament

**DOI:** 10.4102/sajp.v80i1.2079

**Published:** 2024-11-29

**Authors:** John M. Onyancha, Benita Olivier, Joseph M. Matheri, Wallace M. Karuguti

**Affiliations:** 1 Department of Rehabilitation Sciences, School of Medicine, Jomo Kenyatta University of Agriculture and Technology, Kiambu, Kenya; 2 Centre for Healthy Living Research, Oxford Institute of Allied Health Research, Department of Sport, Health Sciences and Social Work, Oxford Brookes University, Oxford, United Kingdom; 3 Wits Cricket Research Hub for Science, Medicine and Rehabilitation, Department of Physiotherapy, School of Therapeutic Sciences, Faculty of Health Sciences, University of the Witwatersrand, Johannesburg, South Africa

**Keywords:** prevalence, incidence, risk factors, rugby injuries, recurrence of injuries, protective gears

## Abstract

**Background:**

Rugby-related injuries are a leading cause of dropout from competitive sports, high insurance compensation, disability and socioeconomic marginalisation. The debilitating effect of these injuries on players may deny them the benefits associated with rugby and can lead to premature termination of a rugby career.

**Objectives:**

To determine the prevalence, incidence and risk factors for rugby-related injuries among male Safari Sevens rugby tournament players in Kenya.

**Method:**

Following ethical approval and clearance, this cross-sectional study was carried out among October 2021 teams of male players in the Safari Sevens rugby tournament. Players (*n* = 113) voluntarily completed Rugby International Consensus Group ‘injury report form’. Descriptive statistics and odds ratios were calculated.

**Results:**

A point prevalence of 47 (41.6%) respondents at pre-tournament was found. The tournament recorded 117.6 player-match hours with an incidence of 33 (29.2%) injuries occurring during competition. In contact phase of rugby match, ‘being tackled’ was associated with a higher number of injuries mostly in the lower limbs. Surface of the playing field was likely to expose a player to injury in pre-competition and during the tournament. Additionally, players who had recurrent previous injuries and oversize gear were more likely to be injured during the tournament.

**Conclusion:**

The study showed competition injury incidence similar to that reported in previous studies. Lower limb injuries were most prevalent, especially the ankle while ‘getting tackled’ during matches.

**Clinical implications:**

There is a need for an algorithm for injury risk assessment, knee and ankle control training protocol, and use of dynamic knee and ankle supports.

## Introduction

Rugby is a highly competitive sport of physical nature, that is beset with injuries. Of particular importance is the rising prevalence of rugby-related injuries that raises public health concern globally. According to Garraway et al. (2000), injuries that occurred in a Scottish rugby union, almost doubled from 27% to 47% in a period of 5 years. Rugby sevens is a variant of rugby union worldwide, played on a standard rugby pitch, where teams comprise seven players who play for 7 min halves with 1 min halftime break (Ross, Gill & Cronin 2014). According to Ross et al. (2014), the Rugby sevens contest by two teams is based on speed and power because of its short duration which most frequently is done in a style of a tournament. Generally, sports of this nature lead to injuries caused by different mechanisms, which affect various body parts (Junge et al. 2009). In rugby, the lower limbs are the most commonly injured body parts, the most prevalent being ankle and knee sprains during competition (King 2006). While head injuries and concussions are also frequent (Constantinou & Bentley 2015; Moore, Ranson & Mathema 2015), these injuries occur mostly during training. Other injuries such as head and shoulder injury may occur in competitions (Fuller et al. 2017).

Whereas researchers attribute the occurrence of most rugby-sports injuries to contact, overuse (Cruz-Ferreira et al. 2018; Junge et al. 2009) and tackling (King 2006), previous research evidence has linked a myriad of other factors with the occurrence of injuries during rugby competition. These include factors such as previous injuries (Cross et al. 2016; Sewry et al. 2019), the lack of protective gears, surface of training and competition (Lanzetti et al. 2017). It also includes factors such as poor physical fitness, inadequate loading, overuse during training for competition (Gabbett & Domrow 2005) and psychological factors (Galambos et al. 2005). Knowledge of factors influencing rugby-related injury occurrence notwithstanding, the consequences of rugby injuries are worrying to sports stakeholders as some result in sports associated disabilities (Brown et al. 2013) and time loss for training or competition (Brooks et al. 2005; Whitehouse et al. 2016). Furthermore, sports injuries-related time loss can result in loss of income, unemployment, difficulties in meeting medical costs, athletes’ availability and poverty among players and their families.

Moreover, the average reported career time-span is reduced by premature career termination of good players (Ristolainen et al. 2012). The career ending to players creates a great concern as it reflects on not only the disabling effect of rugby-sports injuries but also the lack of capacity to reverse the impairments possibly because of the high cost of care. In sub-Saharan Africa including Kenya, there is a scarcity of information regarding rugby-sports injuries despite the Kenyan Rugby sevens becoming a highly popular sport. The purpose of this study was to determine the prevalence, incidence and the risk factors for rugby-sports injuries among the male Safari Sevens rugby tournament players in the year 2020/2021.

## Research methods and design

**FIGURE 1:**
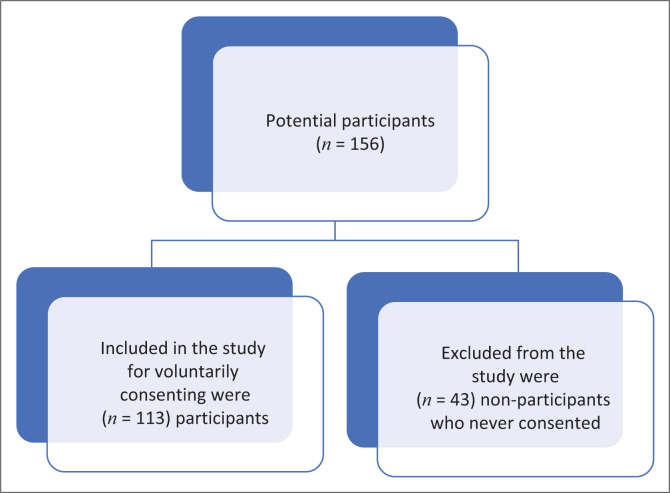
Participants flow chart: Target population of the respondents.

This cross-sectional study was conducted among the Safari Sevens during the 2020/2021 tournament (which was open to international teams) held in Nairobi, Kenya, on 30th and 31st October 2021. There were 12 teams namely; Germany, Spain, South Africa All Stars, Nigeria Stallion, Samurai, Uganda, Red Wailers, Zimbabwe, and four teams from Kenya (i.e., KCB, Chipu 7s, Morans and Shujaaa) with a target population of 156 players (Figure 1). Each team has 13 players (Table 1). The researcher with assistance from team’s physiotherapists collected data in two consecutive stages. In the first stage, the Rugby International Consensus Group (RICG) ‘injury report form’ (Appendix 1) (Fuller et al. 2007) was administered to all consenting rugby players (all male players aged 18 years and above) 2 days prior to the start of match competition to capture information on pre-existing injuries (point prevalence) and the likely risk factors (of the previous injuries). Each respondent’s RICG report form was assigned a unique identifier. In the second stage, during match competition, the respondent’s RICG report forms, comprising the baseline data collected in stage one, were used to record occurrence, and risk factors for injuries among players after clinical confirmation from the team’s medical officers. Injuries were classified by body parts involved.

**TABLE 1:** Respondents’ distribution by teams.

Team	Frequency	%
KCB	12	10.6
Chipu 7s (Kenya U20)	12	10.6
Germany	7	6.2
Spain	11	9.7
South Africa All Stars	11	9.7
Nigeria Stallion	10	8.8
Samurai	3	2.7
Kenya Morans	13	11.5
Kenya Shujaa	10	8.8
Uganda	11	9.7
Red Wailers	4	3.5
Zimbabwe	9	8.0

**Total**	**113**	**100.0**

KCB, Kenya Commercial Bank rugby club; U20, under 20.

Data were entered, cleaned and analysed using Statistical Package for the Social Sciences (SPSS) version 26.0 (IBM, Armonk, New York, United States). Descriptive statistics and the odds ratio (OR) were calculated for players’ age, and playing position, prevalence, and the group-level incidence (injuries/1000 player hours), mean and median of severity of injuries, injured body part (location), type and mechanism (e.g., tackle) of match injuries. According to Marshall and Jonker (2010), descriptive statistics is valuable when the intention is to illustrate the characteristics in a data set including prevalence, incidence and age distribution as was the aim of this study. The ORs were used to determine the risk factors, given that the prescribed RICG report form had a categorical data (with specific factors). The results are presented in summary tables.

### Ethical considerations

The study protocol was approved by Jomo Kenyatta University of Agriculture and Technology (JKUAT) Ethical Review Committee (approval no.: JKU/IERS/02316/0171) and National Commission For Science, Technology and Innovation (NACOSTI) (licence no.: NACOSTI/P/21/13042).

## Results

### Risk factors

One apparent risk was playing position at the time of injury. According to player role when injury occurred, most of the respondents (8, 22.9%) were injured while playing as prop, followed closely by winger (7, 20.0%) and a few as hooker and fly half in equal proportion of 6 (17.1%) each (see Table 2). Regarding those playing backs and forwards positions, the backs had a higher proportion of respondents (20, 57.1%) that were injured compared to forwards (15, 42.9%). In addition, being tackled was associated with a higher number of injuries in contact phase of activity with a *p*-value that was statistically significant at 5% level of significance (*p* ≤ 0.03; 95% Confidence Interval [CI]), and surface of the playing field was more likely (Prevalence Odds Ratio [POR] 2.7; 95% CI, 1.7–3.3: POR 1.6; 95% CI, 1.2–1.9) to expose a player to injury both in pre-competition and during the tournament, respectively (see Table 3 and Table 4). Respondents who had recurrent previous injuries were more likely (POR, 1.28; 95% CI, 1.13–1.81) to be injured during the tournament (see Table 5). Additionally, oversize protective gear was likely (POR 1.9; 95% CI, 1.5–2.3) to expose a player to injury (see Table 3).

**TABLE 2:** Analysis of playing positions at the time of injuries.

Playing position	*n*	%
Prop	8	22.9
Hooker	6	17.1
Fly half	6	17.1
Scrum half	3	8.6
Centre	4	11.4
Winger	7	20.0
Loose head	1	2.9
Forwards	15	42.9
Backs	20	57.1

**TABLE 3:** The odds of risk for injuries pre-competition.

Exposure variables	Response	Respondent pre-competition status				Total		POR	95% CI
		Existing injury		No existing injury					
		*n*	%	*n*	%	*n*	%		
Injured during off-season	No	38	38.8	60	61.2	98	86.7	Ref	-
	Yes	9	60.0	6	40.0	15	13.3	1.4	1.1–1.9
Injured at the start of the season	No	31	34	60	65.9	91	80.5	Ref	-
	Yes	16	72.7	6	27.2	22	19.4	1.6	1.2–1.8
Injured at mid-season	No	29	34.1	56	65.8	85	75.2	Ref	-
	Yes	18	64.2	10	35.7	28	24.7	1.8	0.7–2.1
Playing position at the time of injury winger	No	38	38.8	60	61.2	98	86.7	Ref	-
	Yes	9	60.0	6	40.0	15	13.3	2.4	0.9–2.7
Playing position at the time of injury tight head	No	40	42.1	55	57.8	95	84	Ref	-
	Yes	7	38.8	11	61.1	18	15.9	1.5	0.6–2.2
Tackle, tackling, maul, ruck, lineout, scrum and collision	No	11	17.7	51	82.2	62	54.8	Ref	-
	Yes	36	70.5	15	29.4	51	45.1	2.1	1.8–2.4
Dry natural grass surface	No	13	20	52	80	65	57.5	Ref	-
	Yes	34	70.8	14	29.1	48	42.4	2.7	1.7–3.3
Protective gear was new at the time of injury	No	38	38.4	61	61.6	99	87.6	Ref	-
	Yes	9	64.3	5	35.7	14	12.4	2	0.8–4.6
Protective gear was worn out at the time of injury	No	39	41.9	56	60.2	93	83.8	Ref	-
	Yes	8	44.4	10	55.6	18	16.2	0.3	0.1–2.2
Protective gear was oversize at the time of injury	No	41	41.8	57	58.2	98	86.7	Ref	-
	Yes	6	40.0	9	60.0	15	13.3	1.9	1.5–2.3

CI, confidence interval; POR, prevalence odds ratio; Ref, reference.

**TABLE 4:** The odds of risk for injuries during the tournament.

Exposure variables	Response	Injured during match		Not injured during match		Total		POR	95% CI
		*n*	%	*n*	%	*n*	%		
Tackled was the contact activity	No	56	59.5	38	40.4	94	83.1	Ref	-
	Yes	7	36.8	12	63.1	19	16.8	1.1	1.2–2.1
Tackling was the contact activity	No	57	57.5	42	42.4	99	87.6	Ref	-
	Yes	6	42.8	8	57.1	14	12.3	1.5	0.4–5.2
Collision was the contact activity	No	58	57.4	43	42.5	101	89.3	Ref	-
	Yes	5	41.6	7	58.3	12	10.6	1.5	0.4–5.2
Other contact activity	No	50	53.1	44	46.8	94	83.1	Ref	-
	Yes	10	62.5	6	37.5	16	14.1	1.6	1.1–1.9
In your opinion, has a change in your workload or intensity played a role in the development of your injury or pain?	No	39	81.2	9	18.7	48	76.1	Ref	-
	Yes	10	66.6	5	33.3	15	23.8	0.5	0.2–1.4
During injury the environment was dry natural grass surface	No	46	54.7	38	45.2	84	74.3	Ref	-
	Yes	17	58.6	12	41.3	29	25.6	1.7	1.3–2.2
During injury the environment was artificial tough surface	No	56	57.7	41	42.2	97	85.8	Ref	-
	Yes	7	43.7	9	56.2	16	14.1	1.6	1.2–1.9
Have a full protective gear(s)	No	18	66.6	9	33.3	27	42.1	Ref	-
	Yes	31	83.7	6	16.2	37	57.8	2	0.8–5

CI, confidence interval; POR, prevalence odds ratio; Ref, reference.

**TABLE 5:** Odds of injuries among respondents with existing injuries and recurrent injuries during the tournament.

Category	Variable	Suffered an injury during the tournament				Total		POR	95% CI
		Yes		No					
		*n*	%	*n*	%	*n*	%		
Respondent pre-competition or training status	Existing injury	30	63.8	17	36.2	47	42.0	Ref	-
	No existing injury	49	75.4	16	24.6	65	58.0	0.78	0.8–1.7
Have you ever suffered a similar injury more than once before?	Yes	9	34.6	17	65.4	26	35.6	Ref	-
	No	14	29.8	33	70.2	47	64.4	1.28	1.13–1.81

CI, confidence interval; POR, prevalence odds ratio; Ref, reference.

## Discussion

### Prevalence of rugby-related injuries

The study, conducted in Kenya, showed a high point prevalence (41.6%) of rugby-related injuries, during the Safari Sevens 2020/2021 tournament year. The injuries were mainly of lower limbs (42.5%), with knee (19.1%) being the most common. The high prevalence of injuries was possibly because of high contact activities given the nature of rugby game and the surface condition of match competition. The present findings are similar to those of a study conducted in Argentina among 250 amateur rugby players (senior players from three clubs) that reported an injury prevalence of 52.4%, the lower limb injuries being most common (58.9%) (Tondelli et al. 2022). This corresponds with Muma, Saidi and Githaiga (2012) study conducted among amateur rugby 15s players in Kenya, that showed that 42.1% of rugby-related injuries affect lower limbs with the ankle and foot being the most prevalent. However, our study findings are in contrast to that of the Constantinou and Bentley (2015) study conducted among 626 adolescent rugby players (from school rugby festivals) in South Africa which showed that the head and face injuries were the most prevalent (30%).

### Incidence of rugby-related injuries

The present study established an injury incidence of almost a third of the sample during match competition within a total of 117.6 player-match hours of the tournament. Furthermore, during the match competition, the most prevalent location of injuries was lower limbs with the ankle (18.1%) being most commonly injured part followed by the knee at 12.1%. The incidence of injury was possibly attributed to the surface of competition and risk of recurrence (POR 1.6; 95% CI, 1.2–1.9). These factors are of a great concern because of high economic loss, and training and match playing time loss of the injured players. The present findings are similar to a study conducted by King (2006) in New Zealand among 68 rugby players of which the incidence of injuries was 217.3 per 1000 playing hours, characterised by sprains with more than 45.0% being the lower limb injuries. King (2006) reported knee injuries as the most prevalent at (49.9 per 1000). It is worthwhile noting that the study involved adolescent players. Similarly, Brooks et al. (2005) found almost the same injury incidence in England. According to Brooks et al. (2005), in a follow up of 63 players over a period of 63 weeks of pre-world cup preparation, the incidence was 218 injuries per 1000 player hours. The incidence of injuries is slightly lower in United Kingdom (UK). In a survey conducted among rugby world cup players (*n* = 626) Fuller et al. (2008) found injuries incidence of 83.9/1000 player-match hours, with lower limbs being the most injured. In the Fuller et al. (2008) study, the reported incidence of injuries was lower possibly because of better preparation prior to match competition. Similarly, in a study conducted among 77 players in Portugal, Cruz-Ferreira et al. (2018) found that 60.5% of all injuries were lower limb injuries and the incidence of injuries was 55.84 per 1000 player-match hours. It is worthy to note that the characteristics of the sample (i.e. senior and junior players) in the Cruz-Ferreira et al. (2018) study may have contributed to the slightly lower incidence of injuries. In contrast, Chiwaridzo et al. (2015) study among 275 adolescents (mean age of 16 years) found that the incidence rate was as high as 58.2% with lower limbs being mostly injured (38.6%). The Chiwaridzo et al. (2015) study reported incidence of injuries being higher possibly because the study’s target population was young and whose training, preparation and skills may not have been adequate.

### Risk factors for rugby-related injuries

#### Tackle activities

During the match competition, being tackled was the activity more likely (POR 2.1; 95% CI, 1.8–2.4; POR 1.1; 95% CI, 1.2–2.1) to expose a player to injuries both in pre-competition and during the match competition, respectively. The incidence of injury may be high, probably because players are involved in multiple contacts during the event. According to Cross et al. (2016), during match competition among professional rugby players in the UK (in two seasons) over 50% of the injuries recorded were caused by tackle activities. Further in a longitudinal study lasting 63 weeks of training and prior-world cup preparation for rugby competition in England, Brooks et al. (2005) noted a high incidence (36%) of match injuries occurring during the tackle. Fuller et al. (2017) concurring with the findings found that tackling (44.1%) was the main cause of concussion injuries (risk ratio = 1.84; *p* < 0.001) among elite players (*n* = 639) in Ireland during the Sevens World Series between 2008–2013. Similarly, Constantinou and Bentley (2015) observed that among adolescent players (*n* = 626) in South Africa, most injuries occur during matches with tackle being the most common cause of injury. It is plausible that players that sustain injuries during tackle have poor local muscle stabilisers that give joint stability and sufficient control that muscles provide. According to Retchford et al. (2013), local muscle stabilisers provide suitable joint compression. In addition, a gap player’s tackling skills and resilience when getting tackled have implications for their overall safety.

#### Surface of the playing field

Findings from the present study reveal that the surface of the playing field was more likely (POR 2.7; 95% CI, 1.7–3.3; POR 2.7; 95% CI, 1.7–3.3) to expose a player to injury during a tournament (for both natural grass surface and artificial tough surface). The findings are similar to those reported by Lanzetti et al. (2017) that attributed playing on artificially tough surface to 52% of recorded injuries, among two Italian elite rugby team players (*n* = 40). It is worth noting that injuries were recorded to determine the relationship between ground conditions and risk of injury among a semi-professional team. Similar views were shared by Gabbett, Minbashian and Finch (2007) in Australia, who noted that harder surfaces and grounds are associated with injuries during competitions. Existing research evidence links tough conditions of surfaces used in matches to injury occurrence, which have implications for player(s) performance and likely dropout from training and competition.

#### Previous injuries

Respondents who had recurrent injuries were more likely (POR, 1.28; 95% CI, 1.13–1.81) to be injured during the tournament (see Table 4). The increase in incidence of injuries may be attributed to players’ desire to return-to-active sports before recovery from a previous injury. It may also be because of pressure from coaches wanting the team to perform well or non-adherence to standard guidelines on return-to-competitive sports following treatment and rehabilitation for injuries. Our study findings are similar to those of Quarrie et al. (2001) who found a higher incidence injuries rate among players who had pre-season injuries compared to those without history of previous injuries (RR = 2.41; CI 95% = 1.34–4.32) in New Zealand. Similarly, Whitehouse et al. (2016) reported an incidence of 6.96 per 1000 h, 16% being recurrent injuries among players in an Australian Super Rugby team. This has implications for sports therapists that they should use standard guidelines while formulating advisories on rugby players’ return-to-competitive sports. This has the potential to not only minimise chances of recurrence but also improve the performance of the players.

### The sports protective gear

Importantly, oversize protective gear in particular had higher odds of exposing a player to injury (POR 1.9; 95% CI, 1.5–2.3). Overall, not every protective gear used in rugby absolutely reduces the risk of rugby-related injuries. A study conducted by Marshall et al. (2005) focussed on the role of mouth guards and padded headgear in prevention of injuries. The authors, however, found that the role of mouth guards and padded headgear is to prevent players from lacerations and abrasions, although it has infinitesimal potential for prevention of concussion. In a study (review of videos with case control) conducted on UK Rugby World Cup players (*n* = 547) on the effectiveness of injury prevention using protective gears, Jones et al. (2004) found that headgears can aid in prevention of certain superficial head injuries during the match. However, Jones et al. (2004) noted that the association of headgear with injuries was substantial but not significant statistically (OR = 0.43, 95% CI 0.13–1.19). The lack of properly fitting sports gear has implications for player safety and performance.

## Conclusion

The study established a competition injury incidence similar to that reported in previous studies. Lower limb injuries were most common with ankle being most often injured. The study found an incidence affecting close to 1 out of 3 players, with a higher number of injuries occurring during the contact phase. Surface of the playing field, recurrence of previous injuries and playing gear influence the occurrence of injury. There is implication for design of an algorithm for risk of injury assessment and evaluation of surfaces prior to training and competition, knee and ankle control training protocol as well as prescription of dynamic knee and ankle supports to prevent injuries. Additionally, it is recommended that design of interventional measures to reduce the risk of injuries and future research to compare the incidence of injuries in rugby fifteens to that in rugby sevens be prioritised.

## Appendix 1: Rugby injury measurement tool (Modified)

This tool has three sections, i.e. Section **A:** Collect data on social demographics information, Section **B:** Pre-tournament data collection tool and section **C:** During tournament data collection tool.
